# *Ex Vivo* treatment of coronary artery endothelial cells with serum post-exercise training offers limited protection against *in vitro* exposure to FEC-T chemotherapy

**DOI:** 10.3389/fphys.2023.1079983

**Published:** 2023-02-02

**Authors:** Marie Mclaughlin, Katie L. Hesketh, Sarah L. Horgan, Geraint Florida-James, Matthew Cocks, Juliette A. Strauss, Mark Ross

**Affiliations:** ^1^ School of Applied Sciences, Edinburgh Napier University, Edinburgh, United Kingdom; ^2^ School of Health and Life Sciences, University of the West of Scotland, Lanarkshire, United Kingdom; ^3^ Liverpool John Moores University, School of Sport and Exercise Sciences, Liverpool, United Kingdom; ^4^ School of Energy, Geoscience, Infrastructure and Society, Heriot Watt University, Edinburgh, United Kingdom

**Keywords:** chemotherapy, exercise, endothelium, apoptosis, wound healing, cancer

## Abstract

**Background:** Chemotherapy treatment for breast cancer associates with well-documented cardiovascular detriments. Exercise has shown promise as a potentially protective intervention against cardiac toxicity. However, there is a paucity of evidence for the benefits of exercise on the vasculature.

**Objectives:** This study aimed to determine the effects of chemotherapy on the vascular endothelium; and if there are protective effects of serological alterations elicited by an exercise training intervention.

**Methods and Results:** 15 women participated in a 12-week home-based exercise intervention consisting of three high-intensity interval sessions per week. Human coronary artery endothelial cells (HCAEC) were exposed to physiological concentrations of 5-fluorouracil, epirubicin, cyclophosphamide (FEC) and docetaxel to determine a dose-response. Twenty-4 hours prior to FEC and docetaxel exposure, HCAECs were preconditioned with serum collected pre- and post-training. Annexin V binding and cleaved caspase-3 were assessed using flow cytometry and wound repair by scratch assays. Chemotherapy exposure increased HCAEC Annexin V binding, cleaved caspase-3 expression in a dose-dependent manner; and inhibited wound repair. Compared to pre-training serum, conditioning HCAECs with post-training serum, reduced Annexin V binding (42% vs. 30%, *p* = 0.01) when exposed to FEC. For docetaxel, there were no within-group differences (pre-vs post-exercise) for Annexin V binding or cleaved caspase-3 expression. There was a protective effect of post-training serum on wound repair for 5-flurouracil (*p* = 0.03) only.

**Conclusion:** FEC-T chemotherapy drugs cause significant damage and dysfunction of endothelial cells. Preconditioning with serum collected after an exercise training intervention, elicited some protection against the usual toxicity of FEC-T, when compared to control serum.

## 1 Introduction

Cardiovascular toxicity of chemotherapy is attributed to ∼16% of deaths in those with breast cancer, overtaking breast cancer itself as the most prominent cause of death (Patnaik et al., 2011). There is an ever-growing body of evidence for mechanisms of cancer treatment toxicity, focusing strongly on the heart (Yeh and Bickford, 2009; Cardinale et al., 2015; Cameron et al., 2016). However, vasculature research has received less attention, despite the fact that the vasculature is the first tissue exposed to the treatment upon infusion (Beerepoot et al., 2004).

Chemotherapy treatment is associated with increased circulating endothelial cells (CECs), a biomarker of vascular damage (Beerepoot et al., 2004). This vascular damage is likely responsible for the chemotherapy-induced impairment in endothelial function observed in women with breast cancer, as determined by reductions in flow-mediated dilation (FMD) (Duquaine et al., 2003). Changes in endothelial function are significant, considering that lower FMD scores are associated with CVD presence, including atherosclerosis (Winther et al., 2016) and are also related to future cardiovascular event risk (Cameron et al., 2016). Detriments in vascular health are observed prior to development of cardiac manifestations of chemotherapy toxicity (Ching et al., 2021) and may provide a valuable method for prognostic evaluation of overall cardiovascular risk for those undergoing chemotherapy. Hence, there is a need for the vasculature to be further explored to deliver an understanding of the extent of its role within cardiovascular toxicity of chemotherapy. Specifically, it is suggested that the effects of chemotherapy on vascular endothelial cell health and function are investigated *in vitro* to provide insight into the mechanisms behind the vascular detriments observed *in vivo* (Duquaine et al., 2003; Beerepoot et al., 2004)*.* Therefore, the first aim of this study is to investigate the effects of breast cancer chemotherapy drugs on the endothelium using a human coronary artery endothelial cell (HCAECs) model.

To improve disease-free survival, quality of life and reduce healthcare burden, there is a requirement to tackle chemotherapy-induced cardiovascular toxicity. Intriguingly, exercise has been investigated as a potential therapeutic (Kirkham and Davis, 2015; Lee et al., 2019). So far, effects of exercise interventions during chemotherapy have been promising, demonstrating an attenuation of the usual increase in blood pressure (BP) observed with chemotherapy treatment, subsequently relating to risk reductions of 20% for CVD in breast cancer patients (Sturgeon et al., 2014). These improvements in BP are likely due to, in part, improvements in endothelial cell health and/or function (Hambrecht et al., 2003; Bucciarelli et al., 2017), which may be attributed to exercise-induced alterations in the serological environment, including improved antioxidant (Corbi et al., 2012), anti-inflammatory (Palmefors et al., 2014), and metabolic profiles (Slentz et al., 2016), as well as growth factors (Sapp et al., 2020). Therefore, the second aim of this study was to investigate if exercise training can attenuate any negative impact breast cancer chemotherapy treatment has on endothelial cells *in vitro*, using an *ex vivo* serum exposure model.

## 2 Materials and methods

### 2.1 Study design and ethical approval

The study had 2 experimental arms. Firstly, an investigation into the effects of chemotherapy drugs on endothelial cells *in vitro* was conducted, using current breast cancer chemotherapy drugs. Human coronary artery endothelial cells were exposed to these chemotherapy drugs to determine the effects on cell apoptosis (Annexin V binding to phosphatidylserine and caspase-3 activation (using flow cytometry), as well as endothelial repair using a scratch wound healing assay. Secondly, an investigation into the effects of preconditioning the endothelial cells with serum obtained from women (without a cancer diagnosis) before and after an exercise training regime (Hesketh et al., 2021) on the effects of chemotherapy drugs on endothelial cell health (using the same outcome measures as in arm 1). All *In vitro* experiments were performed in biological triplicate.

This study was approved by the Liverpool Central NHS Research Ethics Committee (approval reference no. 17/NW/0042) and conformed to the Declaration of Helsinki. The study was also approved by Edinburgh Napier University Research Integrity Committee and Liverpool John Moore’s University Ethics Committee.

### 2.2 Participants

Participants (*n* = 15) were referred to the study by their medical general practitioner or self-referred following advertisement in their local area. Inclusion criteria were age 18–65 years with presence of hypertension, anxiety, stress, depression, arthritis, previous heart attack/surgery (not under current investigation), impaired glucose tolerance, overweight/obese (BMI >25 kg m^2^), and/or dyslipidaemia (total cholesterol >5 mmol L^−1^). Participants were excluded for diagnosed cardiovascular and metabolic disease, pregnant or breast-feeding, and those currently carrying an injury.

At baseline, participants (*n* = 15) were aged 51 ± 6 years (35–60 years), with BMI 29.66 ± 4.57 kg m^2^ (22.92–37.31 kg m^2^), $\dot{V}$ O_2Peak_ 20.98 ± 4.99 mL kg^−1^·min^−1^ (15.2–30.3 mL kg^−1^·min^−1^), body fat 38.3 ± 4.7% (30.0%–44.9%), systolic BP 126 ± 15 mmHg (107–168 mmHg), diastolic BP 71 ± 8 mmHg (57–85 mmHg) (Table 1); and had an average of 1 ± 1(Range: 1–3) underlying cardiovascular risk factors including hyperglycaemia, hypertension, obesity, and hyperlipidaemia.

**TABLE 1:** Participant characteristics before and after 12-week exercise intervention.

Characteristic	Pre (*n* = 15)	Post (*n* = 15)	*p*-value
Body Mass Index (kg·m^2^)	30.78 ± 4.81	30.72 ± 4.74	0.774
$\dot{V}$ O_2_peak (ml·kg·min^−1^)	20.98 ± 4.99	23.02 ± 5.79	0.053
Body fat (%)	37.99 ± 5.41	37.79 ± 4.96	0.708
Systolic BP (mmHg)	131 ± 15	126 ± 10	0.194
Diastolic BP (mmHg)	73 ± 8	73 ± 9	0.832
Mean Arterial Pressure (mmHg)	92 ± 10	90 ± 9	0.259

Values shown are mean ± SD.

### 2.3 Baseline measurements

Participants arrived at Liverpool John Moore’s University following an overnight fast, having abstained from caffeine, alcohol, and exercise for 24 h. A sphygmomanometer (Dianamap; GE Pro 300V2, Tampa, Florida) was used to measure blood pressure, using a standard procedure. Dual-energy X-ray Absorptiometry (DXA Hologic QDR Series, Discovery A, Bedford, MA, United States) was used to measure body fat percentage.

### 2.4 Serum collection

Serum tubes (BD Bioscience, United States) were used to collect 14 mL of blood from the antecubital vein. After 30 min, blood samples were centrifuged for 10-min at 900 x *g*. Subsequently, the serum was aliquoted into Eppendorf tubes and stored at −80°C for future *in vitro* experimental work. Total storage time was 10 weeks. Directly before *ex vivo* exposure of serum to the endothelial cells, serum was pooled, creating one pre- and one post-exercise intervention serum sample.

### 2.5 Maximal cardiovascular fitness testing

Peak oxygen consumption ( $\dot{V}$ O_2_peak) was determined using an electromagnetically braked cycle ergometer (Lode Corival, Lode BV, Netherlands). Briefly, participants cycled at 25 W for 3 min, with workload increasing in 35 W increments every 3 min until volitional exhaustion. Breath-by-breath gas analysis determined $\dot{V}$ O_2_peak using the Moxus modular oxygen uptake system (AEI technologies, Pittsburgh, United States). $\dot{V}$ O_2_peak was defined as the highest $\dot{V}$ O_2_ achieved over a 15 s recording period.

### 2.6 Exercise training intervention

The home-based exercise intervention consisted of progressive high-intensity interval sessions, consisting of body-weight exercises, performed 3-times/week for 12 weeks. Participants were advised to follow their usual diet throughout the exercise training intervention. No dietary records were collected. Exercise intervals consisted of two different 30-s bodyweight exercises (chosen from the provided exercise pack, according to self-preference) with no rest in between, followed by 1 min of rest. For week 1 and 2, participants were instructed to perform 4 intervals, with the number of intervals increasing by 1 interval every 2 weeks up to a maximum of 9 interval by week 11 and 12. Throughout all exercise sessions, participants were instructed to wear the provided heart rate (HR) monitor (Polar H10, Polar Electro Oy, Kempele, Finland) and advised to achieve ≥80% of predicted maximum HR (determined by 220-age). To determine adherence and compliance to the intervention, HR data was automatically uploaded to a cloud storage system accessible to the research team. Compliance was defined as the completion of the prescribed number of intervals as well as achieving a HR ≥ 80% HRmax during the session (Hesketh et al., 2021). Adherence was defined as the percentage of weekly prescribed sessions completed, using 3 sessions per week as the maximum (100% adherence). The mean weekly adherence (%) was then calculated (Hesketh et al., 2021). Full methods of how adherence was determined are described elsewhere (Hesketh et al., 2021).

### 2.7 Post-intervention measurements

Post-intervention measurements were undertaken at least 72 h after the final training session and were performed with identical procedures, methods, and timings to baseline measurements.

### 2.8 Endothelial cell culture

Human coronary artery endothelial cells (HCAEC; Sigma-Aldrich, United Kingdom) were cultured in T-75 flasks upon thawing and cultured with MesoEndo Cell Growth Medium (MEGM, product code: 212–500, Sigma-Aldrich, United Kingdom), containing 10% fetal bovine serum and 1% penicillin, 1% streptomycin, 0.1% hydrocortisone, 5% L-Glutamine, 0.2% Endothelial Cell Growth Supplement, 0.1% Ascorbic Acid, 0.1% endothelial growth factor, 0.1% Gentamicin sulfate-Amphotericin-1000, 0.1% heparin. Cells were grown until passage 4, when HCAECs were seeded in 24-well plates with MEGM. HCAECs were grown in MEGM for 24-h before initiation of the *in vitro* experiments.

### 2.9 Endothelial cell chemotherapy exposure

After 24-h, HCAECs were exposed to physiological concentrations of 5-fluorouracil (5-FU), epirubicin hydrochloride, cyclophosphamide, and docetaxel (all Abcam, United Kingdom). The specific concentrations used were based on serum concentrations found in patients after exposure to each of these drugs (5-FU: 1.5 µM (Reigner et al., 2003); epirubicin: 0.006 µM (Danesi et al., 2002); cyclophosphamide: 38 µM (Adams et al., 2014); and docetaxel: 6 µM (Hurria et al., 2006). HCAECs were exposed to FEC combined drugs, with separate exposure to docetaxel, to mimic the common FEC-T administration method prescribed in human early-breast cancer care (Roché et al., 2006). For dose-response experiments, HCAECs were additionally exposed to a dosage 50% below and 50% above the physiological concentrations. For the FEC combined conditions, the incremental dosages of FEC concentrations will be referred to as follows: FEC1 = 5-fluorouracil: 1 µM, epirubicin: 0.003 µM, cyclophosphamide: 19 µM; FEC2 = 5-fluorouracil: 1.5 µM, epirubicin: 0.006 µM, cyclophosphamide: 38 µM; FEC3 = 5-fluorouracil: 2 µM, epirubicin: 0.009 µM, cyclophosphamide: 57 µM. Control samples were exposed to DMSO only, using the same concentrations used in the chemotherapy conditions.

Drugs were washed off at time-points corresponding to the literature findings when serum levels of each drug were diminished in cancer patients. At these time points (5-FU: 3- hours (Reigner et al., 2003); epirubicin: 1- hour (Danesi et al., 2002); cyclophosphamide: 12-h (Adams et al., 2014); docetaxel: 24-h (Hurria et al., 2006); FEC: 4-h), all media was removed from each well, cells washed with PBS and 500 µL fresh MEGM (Sigma-Aldrich, United Kingdom) added to the wells before re-incubating at 37°C in 5% CO_2_ until the appropriate individual analysis time-points.

Flow cytometry analysis time-points were chosen based on the most potent effects of the drugs previously reported in the literature, as determined by levels of cell death when incubated with human breast cancer cells [5-FU: 3 h; epirubicin: 4 h; cyclophosphamide: 3 h (Chow and Loo, 2003), and docetaxel: 48 h (Morse et al., 2005)].

### 2.10 Sample preparation for analysis of drug effects

At the respective time-points, media was removed, cells were washed with 200 µL NaCl, and 100 µL trypsin-EDTA (Gibco, Thermofisher Scientific, United States) solution was used for cell detachment. Cells were transferred to a 96 v-bottom plate and centrifuged at 300 x *g* for 3 min at 21°C. Supernatant was discarded and cells resuspended in 100 µL PBS (Invitrogen, Thermofisher, United States) for analysis by flow cytometry.

### 2.11 Flow cytometry analysis

To determine effects of chemotherapy and serological conditioning on HCAEC apoptosis, a panel of monoclonal antibodies (mAbs) were used. HCAECs were incubated for 30 min at 4°C with anti-CD31-FITC antibody (BioLegend, United States) and anti-Annexin V-PerCP-Cy5.5 antibody (BD Biosciences, United States). For subsequent intracellular staining, endothelial cells were incubated for 20 min at 4°C with 100 µL BD fixation and permeabilization solution (BD Biosciences, United States). HCAECs were then incubated for 30 min at 4°C with anti-cleaved caspase-3-V450 antibody (BD Biosciences, United States). Lastly, HCAECs were washed twice with 100 µL perm wash buffer (BD Biosciences, United States) and resuspended in 200 µL PBS (Invitrogen, Thermofisher, United States).

After performing calibration and fluorescence compensation with CS&T beads (BD Biosciences, United States) and compensation beads (BD CompBeads Set, BD Biosciences, United States), respectively, samples were analysed using the High Throughput System on a 12-colour flow cytometer (FACS Celesta, BD Biosciences, United States). Data were acquired using FACSDiva 6.0 Software (BD Bioscience, United States), with the sample acquisition stopping gate set to 10,000 events.

### 2.12 Flow cytometry gating process

**FIGURE 1:**
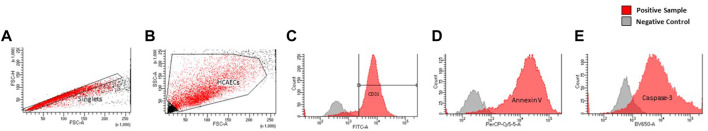
Representative gating strategy of endothelial cells by morphology **(A)**, singlets **(B)**, and CD31 expression as displayed by FITC histogram **(C)**. Histograms of, Annexin-V **(D)**, Cleaved Caspase-3 **(E)** expression are displayed as a function of their respective lasers (PerCp-Cy.5, V450, respectively).

Forward scatter and side scatter dot plots were created to determine the size and morphology of all events, allowing selection of the population of interest. A plot of forward scatter-area and -height was produced to electronically gate on singlet cells (exclusion of doublet cells) and subsequent gating performed on CD31^+^ cells. Histograms of cleaved caspase-3-V450, annexin V-PerCP-Cy5.5 were then created. Representative gating strategies are shown in Figure 1.

### 2.13 Wound healing assay

Passage 4 un-pooled HCAECs (Sigma-Aldrich, United Kingdom) were seeded in 24-well plates with MEGM (Sigma-Aldrich, United Kingdom) either with or without 5% pooled pre- or post-exercise intervention serum and incubated at 37°C in 5% CO_2_ for 24-h, as outlined above. HCAECs were mechanically scraped using a 200 µL pipette to create a vertical wound in the confluent monolayer, washed with 200 µL PBS to remove debris, and 500 µL fresh MEGM (Sigma-Aldrich, United Kingdom) added to each well. HCAECs were then exposed to relevant concentration of chemotherapy drugs as described before.

Cells were imaged using Primovert Axiocam ERc5s microscope (ZEISS, Germany) using ×4 magnification at 0, 3-, 4-, 6-, 12-, 24- and 48-h and analysed using ImageJ (1. x, Java, United States). Rate of wound closure was determined by the area of the wound plotted as a function of time. Area under the curve (AUC) was calculated using known equations (Jonkman et al., 2014).

### 2.14 Serological conditioning of coronary artery endothelial cells

**FIGURE 2:**
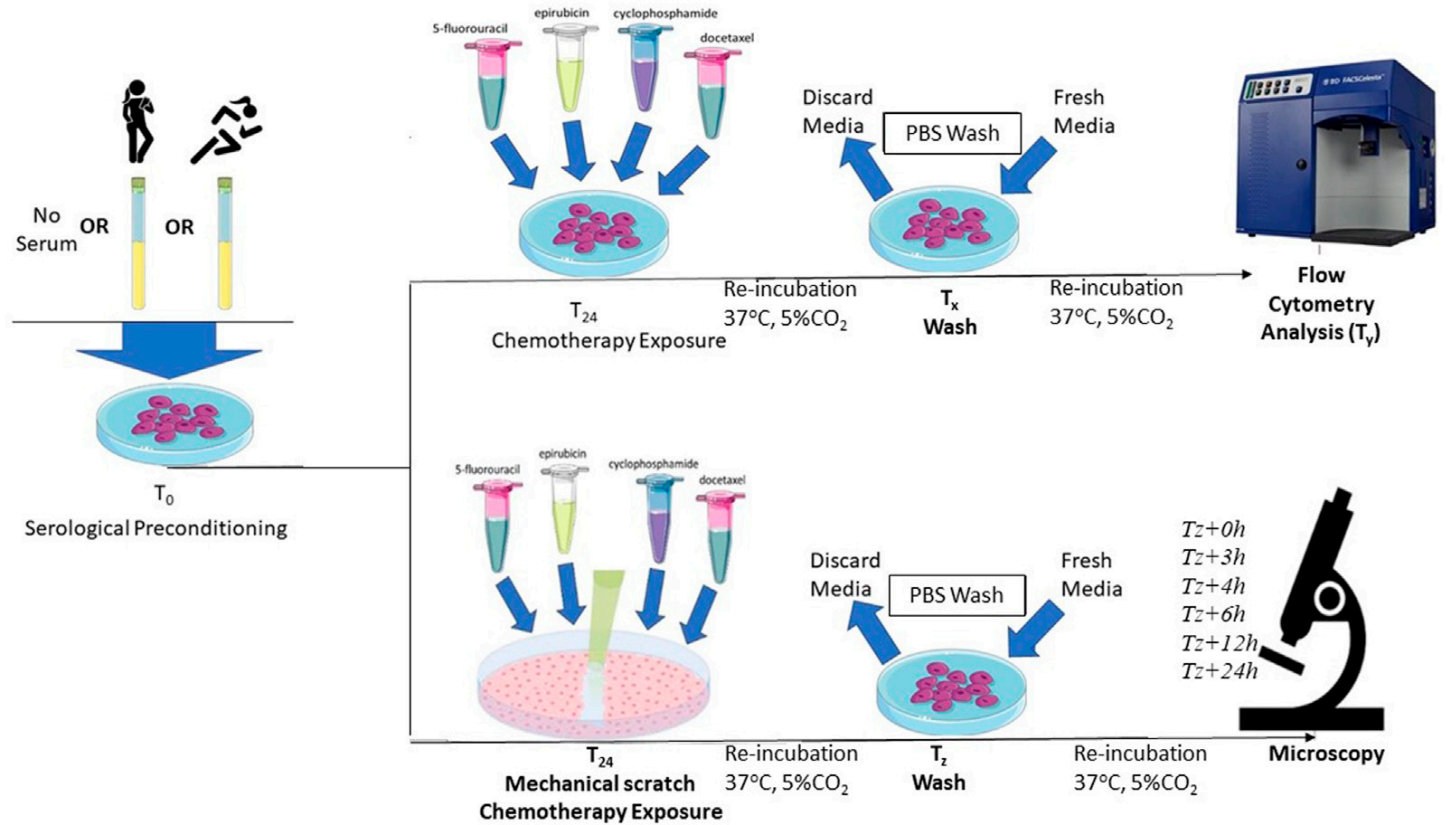
Schematic representation of the protocol for serum preconditioning of HCAECs at 0-h. For flow cytometry analysis, FEC-T chemotherapy exposure occurred 24-h after serum preconditioning. HCAECs were re-incubated and washed with PBS and fresh media added at corresponding time points (T_x_), respective to each individual drug condition. HCAECs were then analysed by flow cytometry at corresponding time points (T_y_), respective to each individual drug condition. For the wound healing assay, FEC-T drugs were added immediately after the monolayer was scratched. HCAECs were re-incubated and washed at corresponding time points (T_z_), respective to each individual drug condition. HCAECs were imaged using Primovert Axiocam ERc5s microscope (ZEISS, Germany) using ×4 magnification at 0-, 3-, 4-, 6-, 12-, 24-, and 48-h.

After 24-h of growth in standard MEGM conditions, the effects of *ex vivo* exposure of HCAECs to serum taken before and after exercise training regime were assessed. HCAECs were cultured with or without 5% pooled pre- or post-exercise intervention serum for 24 h at 37°C in 5% CO_2_ prior to chemotherapy exposure *in vitro*. The serum remained in the media during the chemotherapeutic drug incubation both for flow cytometry measures of endothelial cell apoptosis and caspase-3 activation, as well as prior to the wound healing assay. A visual representation of the full experimental protocol is provided in Figure 2.

### 2.15 Statistical analysis

Dose-response effects of chemotherapy drugs and the between-group difference for serum conditioning on endothelial cell measures were determined using one-way analyses of variance (ANOVA). One-way ANOVAs were also used to calculate between group differences displayed as mean fold change. The effects of the exercise intervention on participant characteristics were determined using a linear mixed model. According to Shapiro-Wilks test of normality, all data was determined to be normally distributed. All analyses were performed using GraphPad Prism Version 9.0.1 (United States) and statistical significance was assumed if *p* < 0.05. All data shown are mean ± standard deviation (SD) unless otherwise stated.

## 3 Results

### 3.1 Participant characteristics

The exercise intervention had no significant effect on BMI, body fat, systolic BP, diastolic BP, or mean arterial pressure (MAP) (Table 1) from pre-to post-intervention. However, $\dot{V}$ O_2Peak_ increased from pre: 20.98 ± 4.99 mL kg^−1^·min^−1^ to post: 23.02 ± 5.79 (*p* = 0.53). The average weekly adherence rate was 75 ± 25% of the prescribed number of sessions over 12-week; and compliance was 82 ± 23%, as determined by the completion of the prescribed number of intervals and achieving a HR ≥ 80% HRmax during the session (Hesketh et al., 2021).

### 3.2 Dose response effects of FEC-T drugs on endothelial cell Annexin-V binding and cleaved Caspase-3 expression

**FIGURE 3:**
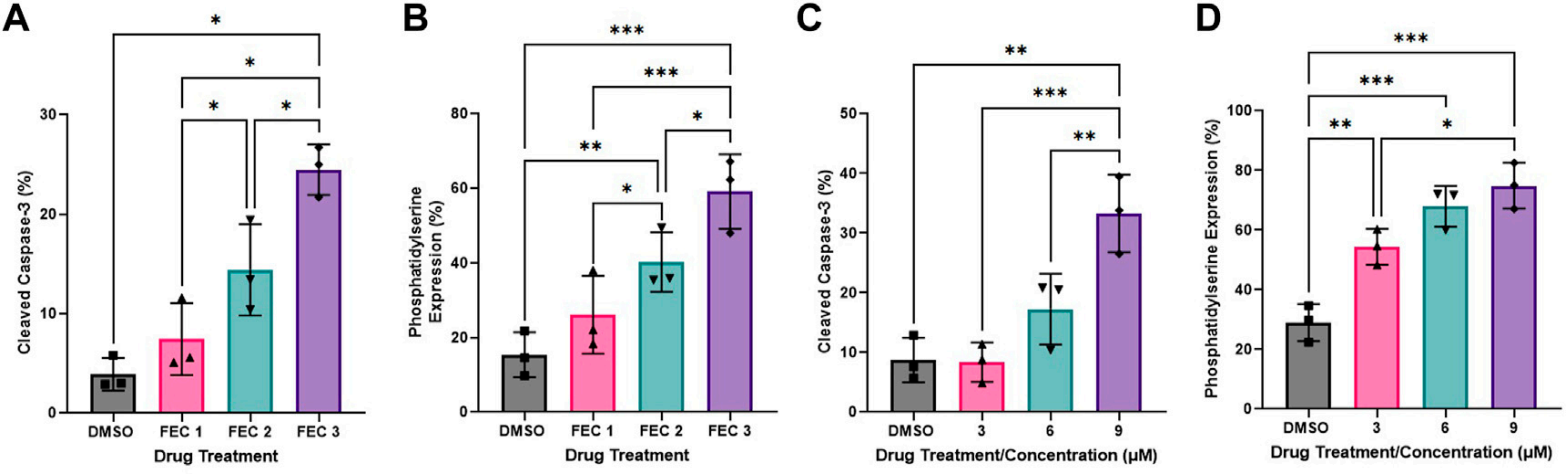
Dose-response effects of combined 5-fluorouracil, epirubicin, cyclophosphamide (FEC; **(A,B)** and docetaxel **(C,D)** on human coronary artery endothelial cells (HCAEC) phosphatidylserine **(A,C)** and cleaved caspase-3 expression **(B,D)** across a range of physiological concentrations at 12- **(A,B)** and 48-h **(C,D)** post-exposure. Data shown are mean ± SEM (*n* = 3). *p < 0.05, **p < 0.01, ***p < 0.001. FEC1 = 5-fluorouracil: 1 µM, epirubicin: 0.003 µM, cyclophosphamide: 19 µM; FEC2 = 5-fluorouracil: 1.5 µM, epirubicin: 0.006 µM, cyclophosphamide: 38 µM; FEC3 = 5-fluorouracil: 2 µM, epirubicin: 0.009 µM, cyclophosphamide: 57 µM.

One-way ANOVAs revealed significant dose-response effects of chemotherapy drug doses on HCAEC Annexin-V binding with exposure to FEC (Figure 3B: DMSO: 8.37 ± 2.09%, FEC1: 26.13 ± 6.03%, FEC2: 40.23 ± 4.59%, FEC3: 59.17 ± 5.76%, *p* < 0.0001). Analysis of Mean Fold Change confirmed significant elevations in Annexin V binding with increasing concentrations of FEC (Mean Fold Change FEC1: 1.83 vs. FEC3: 4.08, *p* < 0.01; FEC2: 2.89 vs. FEC3: 4.08, *p* < 0.05). HCAECs expressed elevated expression of cleaved caspase-3 with exposure to FEC (Figure 3A: DMSO: 3.90 ± 0.95%, FEC1: 7.43 ± 2.09%, FEC2: 14.40 ± 2.65%, FEC3: 24.47 ± 1.47%, *p* < 0.0001) which also occurred in a dose-dependent manner (Mean Fold Change FEC1: 2.19 vs. FEC3: 7.09, *p* < 0.01).

Docetaxel exposure increased HCAEC Annexin V binding to phosphatidylserine (Figure 3D: DMSO: 28.97 ± 6.19%, 3 µM: 54.43 ± 6.00%, 6 µM: 67.90 ± 6.84%, 9 µM: 74.87 ± 7.70%, *p* < 0.0001) with Mean Fold Change analysis revealing that increasing concentrations resulted in Annexin V binding which was significantly different compared to the DMSO control only. Cleaved caspase-3 expression was also increased with exposure to docetaxel (Figure 3C: DMSO: 8.73 ± 3.73%, 3 µM: 8.37 ± 3.27%, 6 µM: 17.23 ± 5.92%, 9 µM: 33.27 ± 6.50%, *p* < 0.0001), with the highest concentration eliciting significantly higher expression than the lowest concentration (Figure 3H: Mean Fold Change 3 µM: 0.97 vs. 9 µM: 4.35, *p* < 0.05).

### 3.3 Effects of FEC-T drugs on endothelial wound healing

**FIGURE 4:**
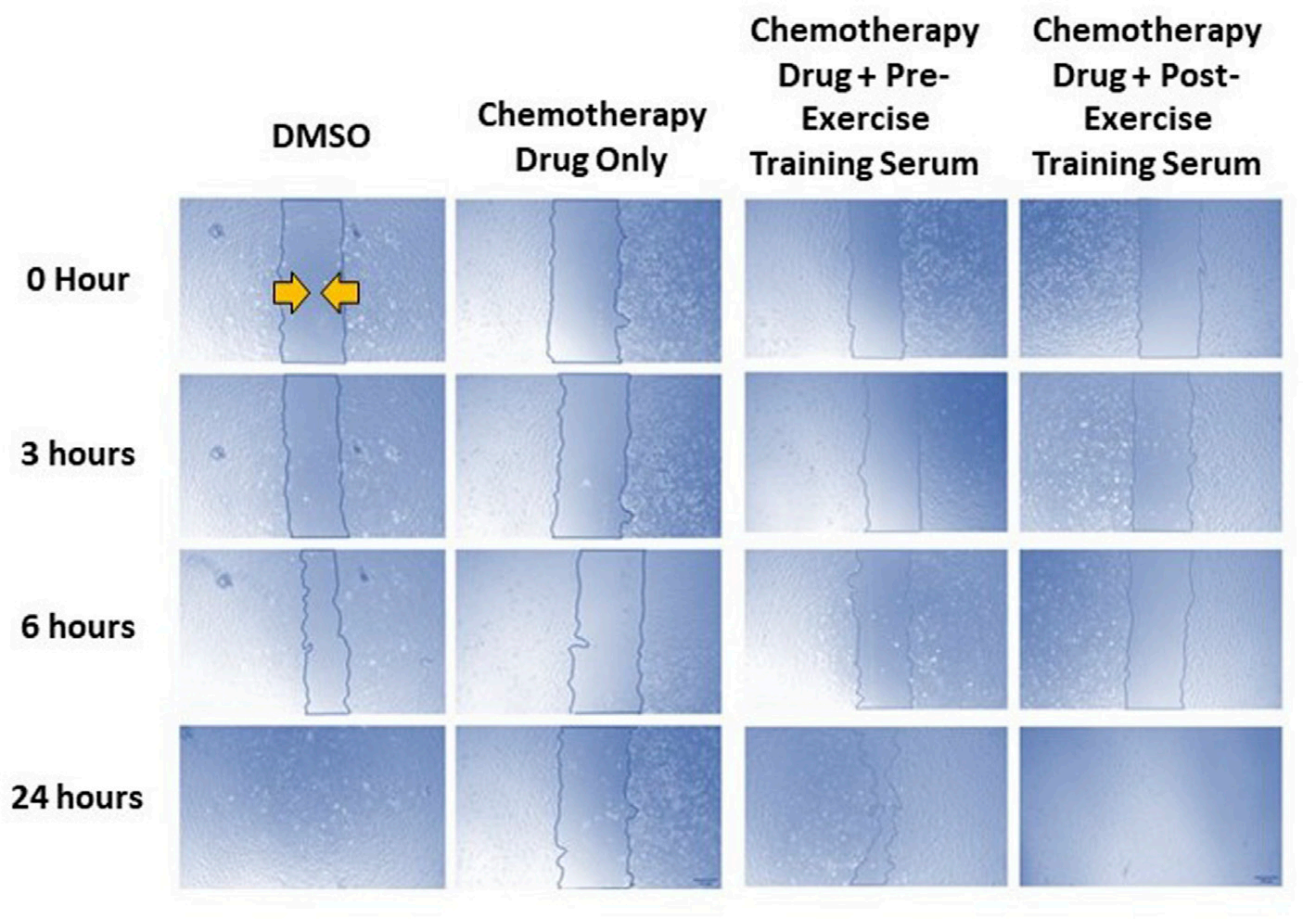
Representative images of human coronary artery endothelial cells (HCAEC) gap closure over time (0-, 3-, 6-, and 24- hours) with and without FEC drug exposure (5-fluorouracil: 1.5 µM, epirubicin: 0.006 µM, cyclophosphamide: 38 µM), and drug exposure with pre- or post-exercise intervention serum conditioning. Images are ×4 magnification. Scale bar is 250 µm.

**FIGURE 5:**
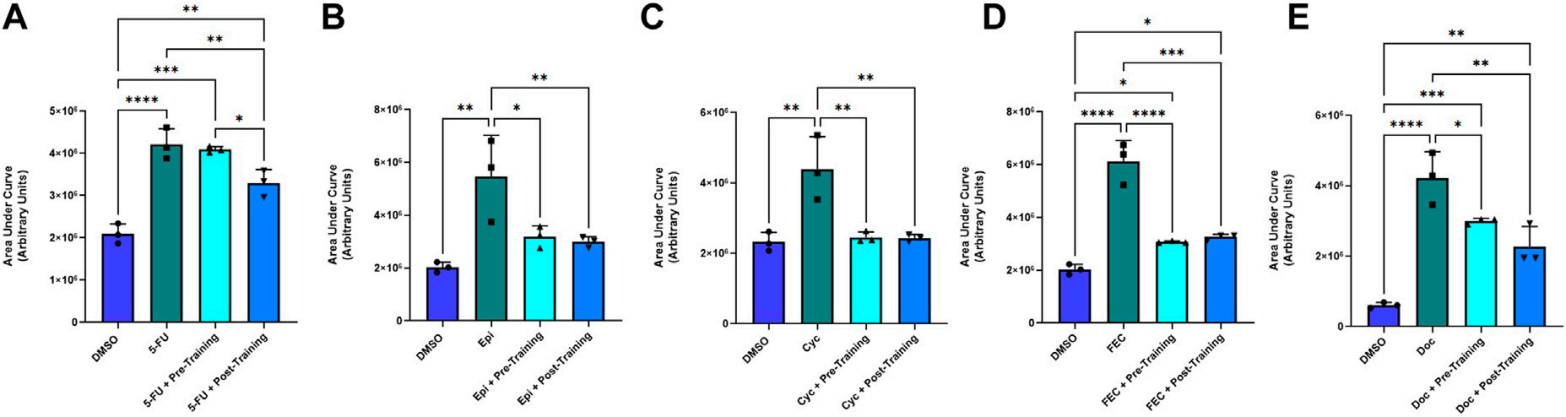
Area under the human coronary artery endothelial cells (HCAEC) gap closure curve with exposure to 1.5 µM 5-fluorouracil **(A)**, 0.006 µM epirubicin **(B)**, 38 µM cyclophosphamide **(C)**, FEC combined **(D)**; 5-fluorouracil: 1.5 µM, epirubicin: 0.006 µM, cyclophosphamide: 38 µM, and 6 µM docetaxel **(E)** treatments with dimethyl sulfoxide (DMSO), drug without serum, pre-training serum with drug and post-training serum with drug. Data shown are mean ± SEM (*n* = 3). *p < 0.05, **p < 0.01, ***p < 0.001, ****p < 0.0001.

Representative images of the wound healing assays are displayed in Figure 4. The rate of wound closure was significantly reduced with all FEC-T drugs individually, as indicated by the increase in AUC (5-FU, *p* = 0.0012; epirubicin, *p* = 0.019; cyclophosphamide, *p* = 0.020; docetaxel, *p* = 0.001) and combined FEC exposure (*p* = 0.048) (Figure 5).

### 3.4 Results of pre- and post-exercise intervention serum preconditioning on FEC-T-induced effects on endothelial cell Annexin-V binding and cleaved Caspase-3 expression

**FIGURE 6:**
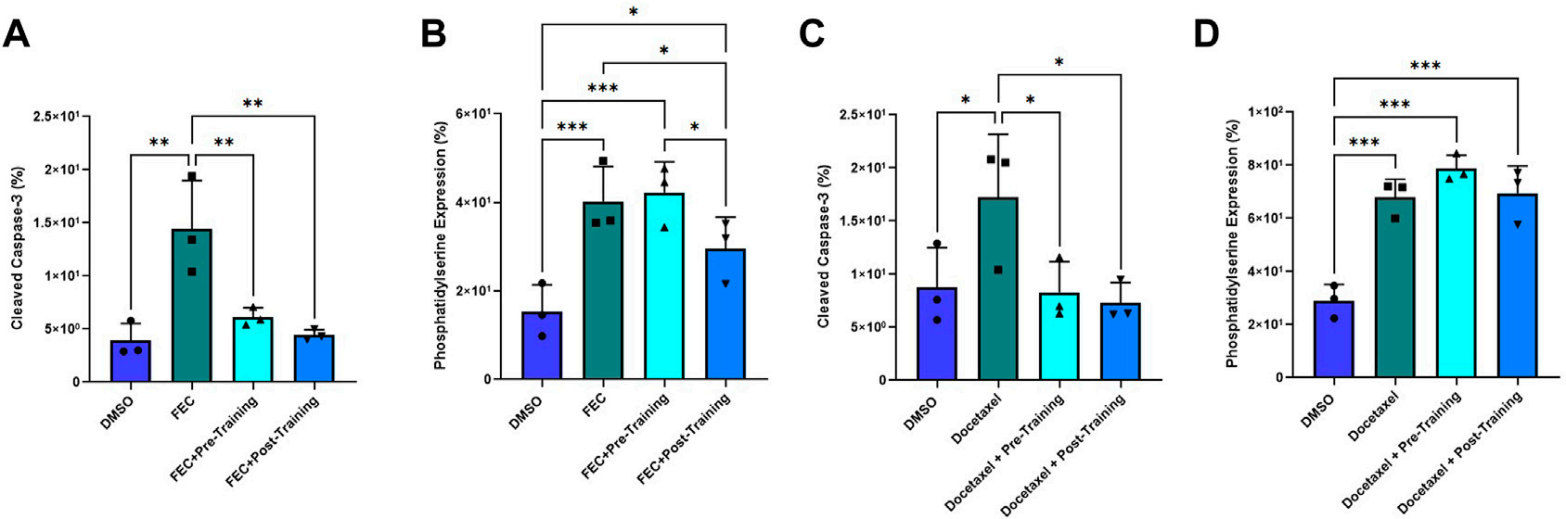
Comparison of pre- and post-exercise intervention serum preconditioning on the effects of FEC **(A,B)**: 5-fluorouracil: 1.5 µM, epirubicin: 0.006 µM, cyclophosphamide: 38 µM) and 6 µM docetaxel **(C,D)** exposure on human coronary artery endothelial cells (HCAEC) phosphatidylserine **(A,C)** and cleaved caspase-3 expression **(B,D)** at 4- and 48-h post-exposure, respectively. Data shown are mean ± SEM (*n* = 3). *p < 0.05, **p < 0.01, ***p < 0.001.

Annexin V binding decreased in the post-exercise serum preconditioning HCAECs compared to pre-exercise training serum when exposed to FEC (Figure 6B). Annexin V binding: pre: 42.23 ± 6.96% vs. post: 29.57 ± 7.09%, *p* = 0.010. Mean Fold Change pre: 1.07 vs. 0.74, *p* < 0.05). Cleaved caspase-3 expression was lower for both serum conditions when compared to the FEC only control (both *p* < 0.01), but there was no significant difference between pre- and post-exercise serum conditions (Figure 6A). Cleaved caspase-3 expression pre: 6.13 ± 0.87% vs. post: 4.43 ± 0.30%, *p* = 0.06. Mean Fold Change pre: 0.47 vs. post: 0.32, *p* = 0.07).

For docetaxel, there were no significant between-group differences for Annexin V binding with serum conditioning (Figure 6D). Both serum conditions elicited significantly lower cleaved caspase-3 expression compared to docetaxel without serum conditioning (Figure 6C). Mean Fold Change pre: 0.51 and post: 0.50; both *p* < 0.05) but there was no significant difference when comparing pre- and post-training serum (Figure 6C). Cleaved caspase-3 expression pre:8.30 ± 2.7 vs. post: 7.33 ± 1.9; *p* = 0.76).

### 3.5 Results of pre- and post-exercise intervention serum preconditioning on FEC-T-induced effects on endothelial wound healing

There was a significantly smaller AUC in favour of the post-exercise training serum condition (Mean Fold Change: 0.789) in comparison to the pre-exercise training serum conditioning group (Mean Fold Change: 0.976, *p* = 0.034) and the chemotherapy only (no serum) condition (*p* < 0.05) when exposed to 5-FU. There were no significant differences for AUC for any other chemotherapy condition when comparing pre-to post-exercise training serum groups, with both serum conditions eliciting lower AUC when compared to epirubicin (both *p* < 0.05), cyclophosphamide (both *p* < 0.001), and FEC (both *p* < 0.001) conditions without serum conditioning (Figure 5).

## 4 Discussion

Our main finding demonstrated that breast cancer chemotherapy drugs exposure resulted in endothelial cell apoptosis and activation of endothelial cell caspase-3 in a dose-dependent manner. Additionally, wound healing was impaired for all chemotherapy drugs, individually, and with combined FEC exposure. Lastly, preconditioning with serum taken after an exercise training regime resulted in modest reductions in FEC-induced HCAEC apoptosis, as well as modestly improved wound healing when exposed to 5-FU, compared to preconditioning with serum from pre-exercise training. No other differences were observed for pre vs. post exercise training serum exposure on FEC-induced changes in wound healing, or docetaxel-induced elevations in phosphatidylserine or caspase-3 expression.

Both FEC and docetaxel treatment conditions resulted in elevated HCAEC phosphatidylserine and cleaved caspase-3 expression, indicating significant vascular toxicity. These findings agree with previous investigations of the effects of chemotherapy exposure on endothelial cell models (Eakin et al., 2020; Bosman et al., 2021) and add to knowledge that endothelial toxicity also occurs in HCAECs exposed to physiological concentrations of FEC combined and docetaxel (Danesi et al., 2002; Reigner et al., 2003; Hurria et al., 2006; Adams et al., 2014). Our *in vitro* observations are relevant and meaningful for patients undergoing chemotherapy, as breast cancer populations elicit significant vascular endothelial damage as shown by 3-fold elevation in CECs after completion of one single treatment cycle (Beerepoot et al., 2004). The damage induced by chemotherapy on the vasculature may have important consequences for CVD development, as without efficient repair, endothelial damage leads to atherosclerotic plaque development and subsequent atherosclerotic CVD initiation and progression (Beerepoot et al., 2004). Development of atherosclerosis has stark cardiovascular consequences, including hypertension and ischaemia, with plaque rupture having potential to cause myocardial infarction and stroke (Cooke and Tsao, 1994; Insull, 2009). Therefore, chemotherapy-induced endothelial apoptosis likely plays a key role in the 9% incidence of cardiovascular events caused by thrombosis in those undergoing anti-cancer treatment (Yeh and Bickford, 2009).

Furthermore, the ability of the endothelium to restore areas of damage is important for protection of the whole cardiovascular system (Michaelis, 2014; Yao et al., 2015), and hence, endothelial wound repair was also investigated in this study. The ability of HCAECs to repair a wound is significantly impaired across all FEC-T chemotherapy conditions. The process of endothelial repair involves detection of a gap in the monolayer caused by damage, which stimulates endothelial cell migration in order to recover the break (Michaelis, 2014). This is essential to prevent atherosclerosis as an unrepaired gap in the endothelial wall leaves opportunity for circulating leukocytes and LDL-cholesterol to be taken up into the tunica intima (Cooke and Tsao, 1994; Insull, 2009). The impedance of wound repair with exposure to each FEC-T condition corresponds to other models of chemotherapy and wound healing, with 5 μg mL^−1^ epirubicin impairing wound healing in HUVECs (Eakin et al., 2020). It is likely that the impairment in wound healing is partly due to high levels of endothelial apoptosis (Yao et al., 2015; Martínez-Limón et al., 2020), as observed in our study also. The chemotherapy-induced impairment in wound healing observed in the current study is likely to have consequences for the whole cardiovascular system as insufficient wound repair increases the risk for plaque development (Cooke and Tsao, 1994; Insull, 2009) and may well contribute to the CVD risk in those undergoing FEC-T chemotherapy treatment (Cameron et al., 2016).

The second arm of this study involved serological preconditioning of the same endothelial cell model to determine if exercise training-induced alterations in the circulating environment can alleviate the effects of chemotherapy exposure. The current findings show that preconditioning HCAECs with serum taken from females at risk of cardiovascular disease after a 12-week home-based exercise intervention elicits a significant level of protection against FEC-T-induced apoptosis, as shown by a 29% decrease in phosphatidylserine expression compared to serum collected before the exercise intervention. This is despite the intervention having no significant effects on other cardiovascular parameters, including $\dot{V}$ O_2Peak_, blood pressure and resting heart rate. This adds to current knowledge that exercise may be beneficial to the vasculature at a cellular level, but it remains unclear whether changes in physiological parameters are necessary to facilitate this change. It must be noted that there was no significant within group effect (pre-vs. post-exercise training) on apoptotic markers with docetaxel exposure. Attenuation of endothelial cell apoptosis due to serological alterations induced by exercise training may provide protection against the usual detriments of chemotherapy for breast cancer which as previously discussed, are likely initiated by endothelial damage (Beerepoot et al., 2004). Further evidence for this comes from investigation of exercise protection of chemotherapy toxicity on cardiomyocytes (Naaktgeboren et al., 2021). A meta-analysis found that preconditioning cardiomyocytes with serum collected after an exercise intervention reduced apoptosis and oxidative stress associated with doxorubicin exposure (Naaktgeboren et al., 2021) likely attributed to by elevated HDL-cholesterol (Durham et al., 2018). As exercise training is a multifaceted therapeutic approach, there are several avenues which should be explored in order to determine the mechanisms of cardiovascular protection against chemotherapy. Other potential mechanism may be a reduction in systemic inflammation, with exercise-induced reduction in pro-inflammatory cytokines, specifically TNF-α (Henrique et al., 2018) and reductions in fasting glucose levels (Slentz et al., 2016) likely inhibiting activation of the apoptotic pathway (Risso et al., 2001; Joussen et al., 2009). The composition of the serum was not investigated within this study due to limitations for the use of the serum, providing an interesting area for future mechanistic research.

Interestingly, there were some promising findings for serological protection of endothelial wound repair. For the 5-FU condition, HCAECs incubated with serum collected post-exercise intervention significantly improved their ability to repair the mechanically inflicted wound. This provides promise that an exercise intervention may stimulate systemic adaptations which elicit vascular protection allowing for more efficient repair when exposed to specific chemotherapy conditions. This study is the first to utilise this serological conditioning approach with wound healing alongside chemotherapy exposure and hence, findings are novel. Investigation of the potential mechanisms of protection are out with the scope of this study. However, a few likely mediators must be considered. Firstly, there is probable involvement of vascular endothelial growth factor (VEGF) as this is a pro-angiogenic factor (Grunewald et al., 2021) which is upregulated with exercise in cardiovascular populations (Gustafsson et al., 2001). As well as VEGF, exercise-induced alterations in circulating cholesterol is another potential contributing factor to the improvements in endothelial wound repair. Levels of serum HDL-cholesterol have been found to increase from pre-to post-exercise training intervention (Palazón-Bru et al., 2021) and has previously been shown to increase HCAEC migration (Kimura et al., 2003). Exercise is a multifaceted therapeutic approach with multiple possible beneficial systemic changes which may protect against vascular toxicity of chemotherapy, including metabolic (Durham et al., 2019), hormonal (Stojadinovic et al., 2012), anti- and pro-inflammatory factors (Palmefors et al., 2014). The specific interactions between serum components and 5-FU remain elusive and were not investigated in the current study. However, the differing effects across the experimental conditions, and the interactions with exercise serum, are likely to be explained by the differing mechanisms of action between each individual FEC-T drug. Future research should take a holistic approach when investigating exercise-induced serological alterations and potential therapeutic targets for attenuation of cardiovascular toxicity in breast cancer patients undergoing these chemotherapy regimens.

### 4.1 Conclusion and limitations

FEC-T chemotherapy exposure induced endothelial cell apoptosis and impaired endothelial wound repair. A 12-week home-based HIIT intervention in ‘at risk’ women resulted in systemic changes which were able to precondition endothelial cells to a state of protection against combined FEC, as shown by reduced apoptotic markers, and an improved ability to recover from mechanical injury when exposed to 5-FU. Reduced endothelial damage and improvement in reparative capacity may well have clinical implications for reducing CVD risk in those exposed to chemotherapy.

Despite some promising results, significant findings did not occur for every outcome measure within every drug condition and therefore, must be considered with the perspective that cardiovascular toxicity is multi-faceted, complex, and interactive. Although hopeful that findings will be relevant to in human studies and that comparisons can be drawn, findings are in fact exclusively *in vitro* and must be considered with modesty. It must also be noted that the participants in this study were non-cancer patients and therefore, there is scope to repeat this study in patients with cancer undergoing an exercise intervention during chemotherapy treatment. Furthermore, a pragmatic approach should be taken to determine the specific components of the serum responsible for the protective effects of exercise training as this provides an avenue for potential discovery of future therapeutic targets to reduce toxicity burden of chemotherapy. The next steps are to perform proteomic analysis of the serum by mass spectrometry to identify key circulating systemic factors potentially contributing to the observed exercise-induced protection. Identified proteins can subsequently be experimentally knocked out to determine causality. This approach will identify key systemic markers which can potentially be pharmaceutically manipulated to reduce chemotherapy toxicity, which is vital for patients who are unable to exercise during their treatment to still obtain the benefits of exercise oncology research. Another limitation which must be noted is the non-physiological flow condition under which *in vitro* experiments were performed. Shear stress is a key factor for regulation and adaptations in endothelial function and health (Gielen et al., 2011). As shear stress has not been mimicked in this cell culture model, it is not possible to provide a conclusion on the potential additional benefits of shear stress. Therefore, future studies should utilise flow conditions as applied elsewhere in the literature (Hattori et al., 2014) to mimic exercise-induced increases in blood flow across the endothelium. Finally, the experimental model utilised in this study has endless potential to explore other malignancies outside breast cancer treatment, and a similar model has been used to explore effects of acute exercise on colon cancer cell growth (Orange et al., 2020). As well as investigating other cancer and treatment types, the model can be used to determine effects of chemotherapy and serum conditioning on other physiological cell types often affected by cancer treatments. Future research in exercise oncology should use the serological conditioning model as a base to explore protection of different exercise interventions against side-effects of several anti-cancer therapies, investigating toxicity within different cell types, as well as investigating protection against tumour progression.

## Data Availability

The raw data supporting the conclusion of this article will be made available by the authors, without undue reservation.

## Abbreviations

5-FU, 5 Fluorouracil; ANOVA, Analysis of Variance; AUC, Area Under the Curve; BMI, Body Mass Index; BP, Blood Pressure; CEC, Circulating Endothelial Cells; CVD, Cardiovascular Disease; DMSO, Dimethyl Sulfoxide; EDTA, Ethylenediaminetetraacetic Acid; FEC, 5 Fluorouracil + Epirubicin + Cyclophosphamide; FEC-T, 5 Fluorouracil + Epirubicin + Cyclophosphamide + Docetaxel; FMD, Flow-Mediated Dilation; HCAEC, Human Coronary Artery Endothelial Cells; HR, Heart Rate; MEGM, Meso-Endothelial Cell Growth Medium; SD, Standard Deviation; TNF-α, Tumour Necrosis Factor-α; VEGF, Vascular Endothelial Growth Factor; $\dot{V}$ O_2_peak, Peak Oxygen Consumption.
